# Electrochemical performance of composite electrodes based on rGO, Mn/Cu metal–organic frameworks, and PANI

**DOI:** 10.1038/s41598-021-04409-y

**Published:** 2022-01-13

**Authors:** Quoc Bao Le, Thanh-Huong Nguyen, Haojie Fei, Constantin Bubulinca, Lukas Munster, Nikola Bugarova, Matej Micusik, Rudolf Kiefer, Tran Trong Dao, Maria Omastova, Natalia E. Kazantseva, Petr Saha

**Affiliations:** 1 University Institute, Nad Ovčírnou 3685, 760 01 Zlin, Czech Republic; 2 Conducting Polymers in Composites and Applications Research Group, Faculty of Applied Sciences, Ton Duc Thang University, Ho Chi Minh City, Vietnam; 3 NTT Hi-Tech Institute, Nguyen Tat Thanh University, Ho Chi Minh City, 72820 Vietnam; 4 Polymer Institute, Slovak Academy of Science, Dubravska cesta, 9, 845 41 Bratislava, Slovakia; 5 Division of Modeling Evolutionary Algorithms Simulation and Artificial Intelligence, Faculty of Electrical & Electronics Engineering, Ton Duc Thang University, Ho Chi Minh City, Vietnam

**Keywords:** Energy, Supercapacitors, Conjugated polymers

## Abstract

Benzendicarboxylic acid (BDC)-based metal–organic frameworks (MOFs) have been widely utilized in various applications, including supercapacitor electrode materials. Manganese and copper have solid diamond frames formed with BDC linkers among transition metals chosen for MOF formation. They have shown the possibility to enlarge capacitance at different combinations of MOFs and polyaniline (PANI). Herein, reduced graphene oxide (rGO) was used as the matrix to fabricate electrochemical double-layer SCs. PANI and Mn/Cu-MOF's effect on the properties of electrode materials was investigated through electrochemical analysis. As a result, the highest specific capacitance of about 276 F/g at a current density of 0.5 A/g was obtained for rGO/Cu-MOF@PANI composite.

## Introduction

Humankind and the environment are highly dependent on the type of energy consumed. Environmental pollution from traditional fuel sources has caused the world to develop renewable energy sources such as supercapacitors (SC), batteries, and hybrid systems^1–5^. Although they differ from each other both in function and in the application, SCs surpass batteries in energy density and capacity^6,7^. These characteristics have been achieved through multicomponent use of nano-materials with a controlled structure^8–10^.

One way to increase the electrode material's capacity is to use carbon materials as a matrix for charge accumulation via an electrochemical double layer^11,12^. Thus, adding specific functionalities in the carbonaceous system to create preeminent composites for electrode system withdraws considerable attention in the development of energy storage devices^13^.

Choi et al. reported that MOFs could be integrated into supercapacitor SC devices due to metal oxide and organic constituents. Their results showed that MOFs could provide sufficiently high capacitance and durability for SC electrodes^14^. Although MOFs have lower electrical conductivity than carbon-based materials, they can be used as components of graphite carbon materials to make active electrode materials of SCs^15^. MOFs are attractive candidates to meet the requirements of the following generation energy storage technologies. They can easily combine carbon-based materials to form the high-working performance composite applied for SCs’ electrodes. The strategies of combining MOFs and other carbon-based materials with conducting polymers can help increase the energy storage capacitance^16–18^.

In the previous study, we synthesized the rGO and Zn-MOF composites combined with PANI in different forms^19^. As a result of the study, we found that rGO/Zn-MOF aerogel exhibits much better electrochemical performance after in-situ modification with PANI.

**Figure 1:**
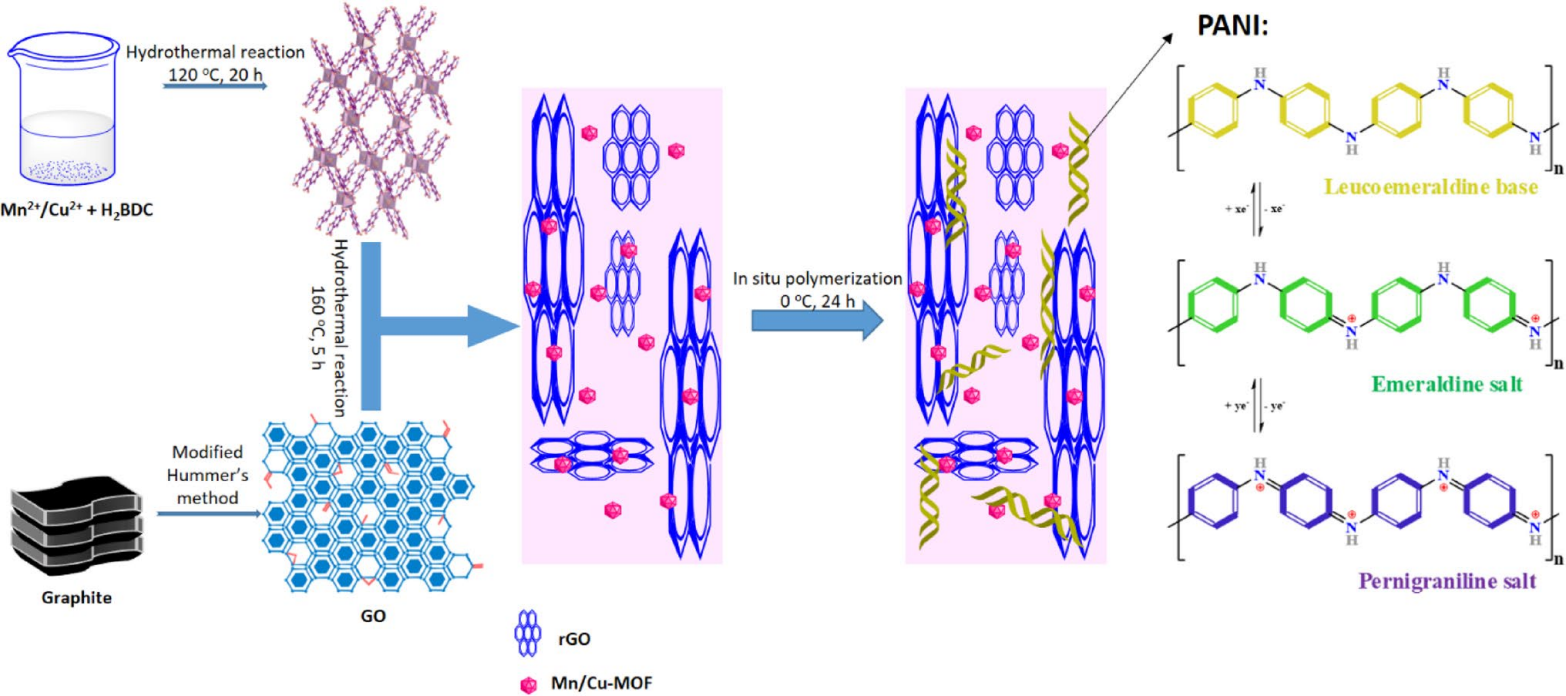
The fabricated supercapacitor based on rGO, Cu/Mn-MOF, and PANI.

In this study, the electrodes for SCs were fabricated in hybrid composites made of rGO, MOFs based on Mn or Cu, and PANI. The structure of electrodes obtained is depicted in Fig. 1. The MOFs can enhance electrolyte ions' adsorption/desorption behaviors during EDLC charging/discharging of SCs and give potential opportunities in turning their electronic and electrochemical properties via modifying their metal center and organic linkers^17^. The changes in organic linkers may lead to a shift in their crystalline structures and electrochemical properties^6^. MOF powders can serve as a stable and underlying conductive network with high electrical conductivity and improved cycling stability in hydrogel materials^20^. Based on the previous results, we propose MOF synthesis using different metal ions. Hence, it can enhance electrolyte ions' adsorption/desorption behaviors during EDLC charging/discharging of SCs. The MOFs give potential opportunities in turning their electronic and electrochemical properties via modifying their metal center and organic linkers^17^. The idea of this study is aiming at the structure of MOF based on Cu^2+^, and Mn^2+^ developed from our previous study.

## Experimental

### Materials

Synthetic graphite powder (< 20 μm), potassium permanganate (KMnO_4_ ≥ 99.0%), ammonium persulfate ((NH_4_)_2_S_2_O_8_, ≥ 98.0%), aniline, Manganese (II) chloride tetrahydrate (MnCl_4_·4H_2_O), Copper (II) nitrate trihydrate (Cu(NO_3_)_2_·3H_2_O), terephthalic acid (H_2_BDC, 98%), *N*,*N*′-dimethylformamide and perchloric acid (HClO_4_, 70%) were products of Sigma-Aldrich. Sulfuric acid (96%), hydrogen peroxide solution (30%), and all solvents were purchased from local companies PENTA and VWR Chemicals. All chemicals were used without any further purification.

### GO preparation

Graphene oxide (GO) was prepared according to the modified Hummers’ method. Firstly, a four-neck flask containing 3 g of graphite powders and 3 g of KNO_3_ were put in an ice bath. Then, 150 mL H_2_SO_4_ (96%) was slowly added to the flask, and the suspension was stirred for 15 min. Next, 18 g of KMnO_4_ was slowly added to the mixture under continuous stirring. The mixture was then stirred at 35 °C for 1 h. After that, the suspension was put into an ice bath, and 200 mL of deionized (DI) water was dropped slowly into the mixture to reach room temperature. An additional 100 mL of DI water was added before the mixture was re-heated to 90 °C. Then, 40 mL of hydrogen peroxide (30%) was slowly poured into the mixture and stirred for 30 min. Subsequently, the mixture was cooled down to room temperature, washed with DI water, and centrifuged until its pH was approximately neutral. The generated GO suspension was kept in cold conditions for further experiments.

### Synthesis of Mn-MOF and Cu-MOF

Mn-MOFs were synthesized following a typical procedure described previously with modification^21^. In brief, MnCl_2_·4H_2_O (1.187 g, 6 mmol) and H_2_BDC (0.199 g, 1.2 mmol) were dissolved in 15 mL of dimethylformamide (DMF). The mixture was stirred and sonicated until complete dissolution. The mixture was then transferred into a Teflon-lined stainless-steel autoclave, heated up to 120 °C for 20 h then cooled to room temperature. The crystallites were collected and washed several times with methanol and DMF before being centrifugated at 4000 rpm for 30 min. The obtained residue was activated by removing the solvent under vacuum at 100 °C over 12 h to produce Manganese-1,4-Benzenedicarboxylate (Mn-BDC). The preparation of Copper-1,4-benzenedicarboxylate (Cu-BDC) followed the same procedure, but Cu(NO_3_)_2_·3H_2_O (1.45 g, 6 mmol) was used instead of MnCl_2_.4H_2_O.

### Synthesis of rGO-MOF composites

Two different composites were prepared. Accordingly, rGO/Mn-MOF aerogel (M1) was synthesized using a one-step hydrothermal co-assembly method^22^. A mixture of 0.02 g Mn-MOF and 5.589 g GO suspension (17.89 mg/mL) was sonicated for 60 min. The content was then transferred to an autoclave, heated to 160 °C for 5 h, and then cooled to room temperature. The hybrid hydrogel was collected, washed several times with DI water, and kept in 10 mL of DI water. A similar procedure was applied in preparing rGO/Cu-MOF (M2), but 0.02 g Cu-MOF was used instead of Mn-MOF. All hybrid composites were freeze-dried to obtain M1 and M2 composites.

### Modification of rGO-MOF composites by PANI

Both rGO-MOF composites supported with PANI, M1, and M2 were modified via in-situ polymerization of aniline^23^. To this end, a piece of synthesized graphene hydrogel was immersed in 50 mL of DI water in a 100 mL beaker. Then 1.5 mL of aniline was added. Hence, the solution was kept for 2 h at room temperature before the hydrogel piece was taken out. This step was repeated before a piece of hydrogel was immersed in a 50 mL solution of perchloric acid (6.8%) for 18 h. After that, the sample obtained was immersed in perchloric acid solution (9.2%, 22 mL) and kept at 0 °C for 1 h. Then, the ammonium persulfate (17.5 mM, 10 mL) solution was dropped slowly into a perchloric acid solution under continuous stirring. After that, the solution was kept stabilized at 0 °C for 24 h to polymerize. The samples were taken out and were rinsed with DI water and then freeze-dried to obtain rGO/Mn-MOF@PANI (M1P) and rGO/Cu-MOF@PANI (M2P).

### Characterization techniques

We conducted the characterizations using the methods of our previous studies^19,22^. Mn-MOF and Cu-MOF's crystal structures were analyzed via X-ray powder diffraction (XRD) patterns employing a Cu–Kα radiation source from a D8 Advance Brucker powder diffractometer. The chemical composition was identified by ATR-FTIR spectroscopy by using Nicolet iS10 (Thermo Scientific) equipped with an ATR sampling accessory with a Ge crystal plate. The pore morphologies were characterized by a NANOSEM 450 (FEI, USA) scanning electron microscope (SEM) operated at 5 kV under 90 Pa pressure. The prepared samples were also investigated by transmission electron microscopy (TEM) employing the JEOL JEM-2100 electron microscope (JEOL, Japan) operated at 160 kV accelerating voltage. The samples were ultrasonically dispersed in water (0.5 wt%), drop-cast onto formvar coated 300 mesh copper grids, and gently dried.

The determination of C, H, N was conducted by the FLASH method. The content of elements in the samples was determined by the EDX-XRF method (Energy ray dispersion spectroscopy—X-ray fluorescence), which was based on the elemental analysis of X-ray diffraction (energy) materials. Each element has a different X-ray energy radiation by type and quantum of X-ray. Radiation was determined by the type and amounts of elements in the sample.

To analyze the chemical composition and binding energy of the composites obtained, X-ray Photoelectron Spectroscopy (XPS) was applied. The measurements were carried out by using the photoelectron spectrometer Hermo Scientific K-Alpha XPS system (Thermo Fisher Scientific) with a monochromatic Al Kα source, pass energies of 200 eV (step size 1.0 eV) and 30 eV (step size 0.1 eV) for the survey and high-resolution spectra, respectively.

To further identify Mn and Cu's components, we used the EDX method to determine them. The content of elements in the samples was determined by EDX-XRF method (Energy ray dispersion spectroscopy—X ray fluorescence), which is based on elemental analysis of X-ray diffraction (energy) materials. Each element has a different X-ray energy radiation by type and quantum of X-ray. Radiation is determined by the type and amount of elements in the sample. The method is suitable for determining elemental representation in matrices in powder, liquid, and solid form; it is a non-destructive method (the study of material surfaces). The amount of elements analyzed is evaluated in weight percent (% m/m). Powered samples in special cups were placed inside the instrument at the appropriate sample to the autosampler. The content of the elements was determined using an Energy Dispersive X-ray Spectrometer (Thermo Scientific, ARL Quant X). The samples were analyzed in a specially selected method: any sample Helium in Quant program.

### Fabrication of working electrodes and their electrochemical testing

The electrochemical test was performed following our previous studies^19,22^. A slurry containing the crushed composite using a solution of PTFE (10% of total mass) in 1 mL of ethanol was prepared. Hence, the slurry was coated as a circle of 0.25 cm radius on a titanium mesh and then compressed. The as-prepared electrodes' electrochemical performance was investigated using cyclic voltammetry (CV), galvanostatic charge–discharge tests, and electrochemical impedance spectroscopy (EIS) techniques on a potentiostat Autolab PGSTAT-128 N at room temperature.

Electrochemical measurements were performed in a three-electrode cell using a working electrode, a platinum wire with a high area dimension electrode, and an Ag/AgCl reference electrode. The measurements were carried out in an aqueous solution of two different electrolytes of 1 M H_2_SO_4_ at room temperature.

The prepared materials' cycling stability was measured in a two-electrode system by BioLogic battery cyclers (BCS-810) at a current density of 1 A/g for 5000 times. In the two-electrode system, the two same electrodes were compressed in a Swagelok cell and separated by a piece of supercapacitor separator (NKK-MPF30AC).

## Results and discussion

This study also used the 3D carbon-based materials (CBM) synthesized from 2D graphene oxide. The 3D substrate materials have higher porosity and specific surface areas. However, they also have the drawbacks of low conductivity and poor hydrophilic^13^. Due to the sp^2^ hybridization inside the structure, 2D CBMs have better electrochemical properties and good electrical conductivity and can be widely composed with CPs in widespread application in SCs. However, the 2D CBMs can accumulate easily, such as GO, during the synthesis processes^13^.

Furthermore, during the working process in the ambient conditions, the layer made of 2D CBMs can be easy peeled off from the electrode surfaces. However, their composites may help to hinder and diminish the self-strip phenomenons. To increase the working performance of the supercapacitor electrode materials, MOFs were used. MOF powders can serve as a stable and underlying conductive network with high electrical conductivity and improved cycling stability. Such composite materials can help to prepare the next generation of electrochemical energy harvesting and storage devices with long cycle life^20^. Changing the porosity of MOFs required different synthesis conditions. Every changing factor will lead to a difference in the morphology of the MOFs.

Our composites were made of the rGO matrix connecting the other components inside its structure. Moreover, during the polymerization process, the matrix absorbed aniline inside the solution by its porous structure. The composites have the scaffold of rGO sheets on which PANI and MOFs adhered. In contrast to the rGO matrix, MOF has a crystalline structure. Their surface is smooth, as can be seen from the SEM picture, and aniline cannot attach. If we synthesize MOF@PANI using the same process applied for M1P and M2P, PANI will be synthesized separately from the structure of MOF during the polymerization process. It means that there is no composite MOF@PANI can be made. We can only get the two different compounds PANI and MOF separately after the reaction. Hence, the MOF only cannot form the electrode materials because of their smooth surface. In the previous study, we attempted to make the electrode containing the MOF only. However, the fabrication is not stable, and the electrode will be destroyed after drying.

The purpose of this study is the application of rGO composite as electrode materials. As we mentioned in the introduction part, this study focused on improving the operating performance of rGO and MOF further investigating our previous study. rGO is well known as the typical materials for SC; however, they also contain drawbacks such as low specific surface areas. The addition of rGO and PANI to the composite aims to improve the electrochemical properties of the electrode’s materials. Their application as SC electrode materials can be found in the litteratures^4,24^.

Our group had published a paper related to the effect of the concentration of aniline or pyrrole^25^. We reported that the aniline or pyrrole monomer concentrations (3% and 6%) did not affect the polymers' products. According to those results, we chose the standard concentration 3% of monomer solution in this study. The relative amounts of rGO: MOF chosen in this study were optimized from our previous publication. We did optimizations and found in the composite, if the amount of MOFs increases after the hydrothermal reaction, the large amount of MOF powders would be superfluous and precipitated inside the solution. Hence, the amount of MOF inside the composite cannot be predicted precisely.

### XRD and FTIR characterizations

**Figure 2:**
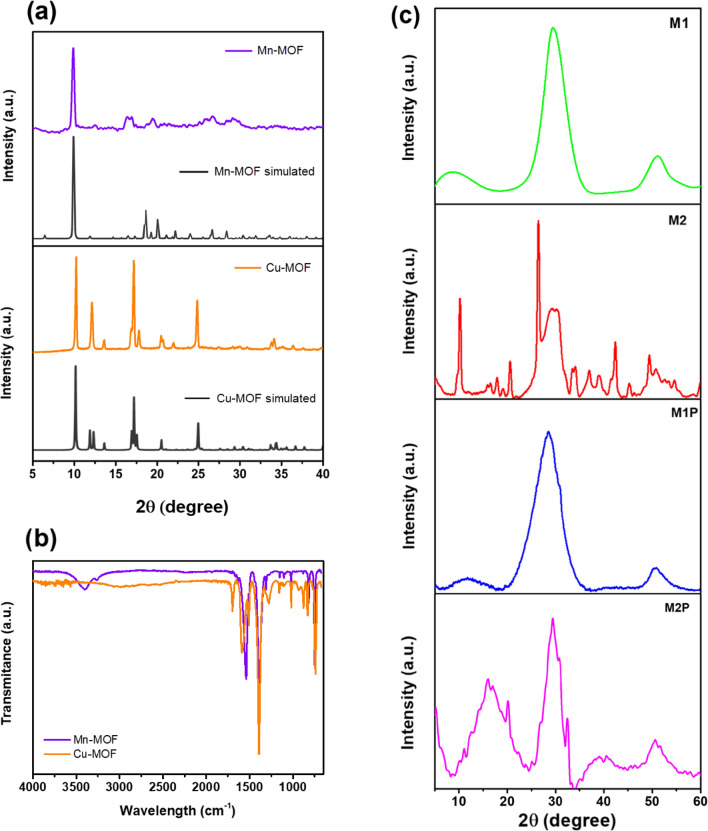
(**a**) XRD and (**b**) IR spectra of Mn-MOF and Cu-MOF; and (c) the XRD spectra of all composites.

The MOFs materials were analyzed by different methods to identify their structural and chemical features. Characteristic XRD peaks were found at 10.2°, 12.1°, and 24.9° for Cu-BDC (MOF) and 10.1°, 14.7°, and 24.5° for Mn-MOF (Fig. 2a). The observed XRD spectra are in good agreement with the simulated one (black line). The XRD pattern of Cu-MOF was compared with the Cu-BDC XRD simulated in the literature reported by Silva et al.^26^. The diffraction patterns of Mn-BDC are consistent with the simulated XRD pattern and compared with the literature (monoclinic crystalline framework patterns of MnO_6_with octahedral geometry, Mn_3_(1,4-BDC)_3_(m-DMF)_2_)^27^. Hence, XRD spectra was also compared with the simulated Mn-BDC (MOF) XRD reported by Asghar et al.^28^. It can indicate that the Mn-BDC (MOF) and Cu-BDC (MOF) were successfully synthesized. According to previous studies, the XRD data suggest that solvothermal synthesized Mn/Cu-MOFs contain diamond-shaped channels^22,29,30^. Besides, the crystalline structure of Mn-MOF detected via XRD depends on the solvent inside the structure. The Mn-MOF structure was changed during the heating vacuum process while DMF molecules were removed from the skeleton structure. It changed the 3D crystalline dimension distance inside the Mn-MOFs after the thermal activation. As a consequence, the Mn-BDC (MOF) diffraction peaks were attenuated^31^.

The XRD spectra of all composites are shown in Fig. 2c. After the hydrothermal reaction, the XRD spectra of Mn-MOF in M1 and M1P showed the most substantial peak at 29.5°. Due to the attenuation of Mn-MOF, their peaks were covered. However, the peaks at 9.2° showed that the existence of Mn-MOF in the composites. In contrast, M2 and M2P show high crystallinity in their XRD spectra. Those spectra showed the stability of the crystalline structure of MOFs in the composites after the synthesis processes.

The Fourier transforms infrared (FTIR) spectra of Mn/Cu-MOF’s are shown in Fig. 2b. As observed, the signal at 3362 cm^−1^ displays the –OH stretching; the peak at 1588 cm^−1^ relates to the asymmetric vibration of –COO. The asymmetric and symmetric stretch of carboxylate groups corresponds to the peak at 1392 cm^−1^. The peaks at 1159 cm^−1^, 1107 cm^−1^, and 1019 cm^−1^ correspond to C–C vibration. Those results also confirmed that Mn/Cu-MOFs were successfully synthesized via hydrothermal reaction.

**Figure 3:**
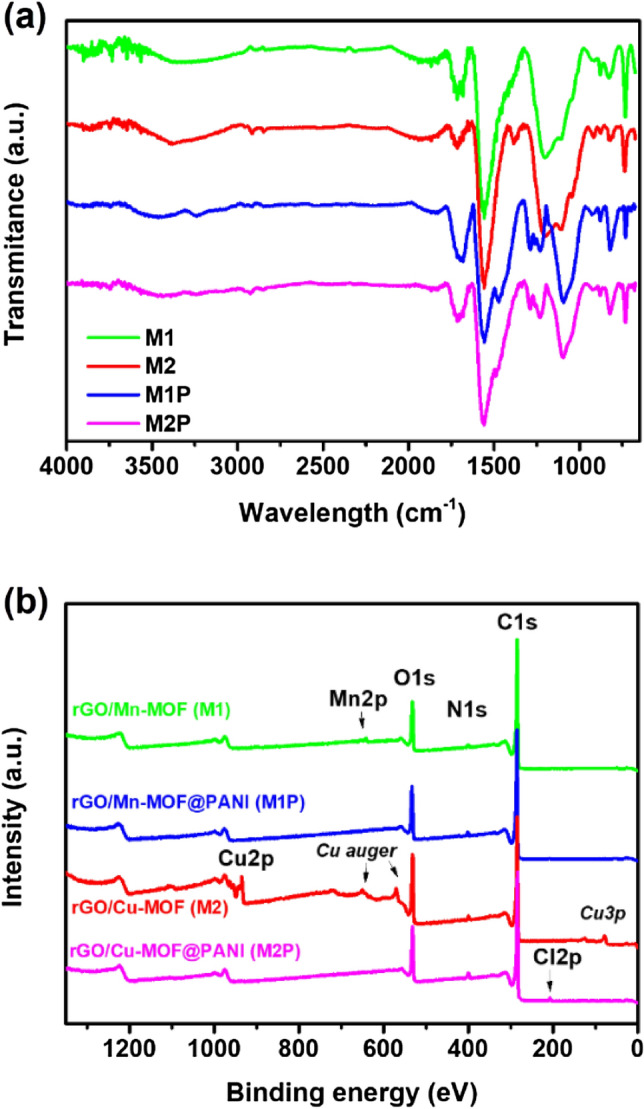
(**a**) FTIR spectra; and (**b**) XPS survey spectra of M1, M2, M1P, and M2P.

Figure 3a displays the IR spectra of composites. The composites were formed after the reduction of GO to make rGO sheets via hydrothermal reaction. After the hydrothermal reaction, the GO is reduced to rGO. Hence, carbonyl groups (C=O) are reduced to methylene (–CH_2_–). The peaks at 2983 and 2901 cm^−1^ in all spectra show the symmetric and asymmetric stretching vibration of –CH_2_–, respectively^32^. The stretching vibration of the C=C plate of samples depicts the peak at 1580 cm^−1^. The sp^2^ carbon stretching vibration peaks of C=O due to the carboxylic group site at the edge of GO are displayed at 1714 cm^−1^. At 1565 cm^−1^, the strong peaks represent the aromatic C=C in-plane vibrations in the rGO sheet. Hence the peaks at 1203 and 1095 cm^−1^ displayed the C–O stretching vibrations and the carbonyl groups leftover after reduction^33,34^. Due to the resemblance in the aromatic structure, the FT-IR spectra of those composites appeared similar to each other. However, in M1P and M2P, the band at 1232–1289 cm^−1^ can be assigned to the π-electron delocalization induced in the polymer through protonation or C–N–C stretching vibration. Furthermore, the peak at 1232 can show the C–N^+^ stretching vibration in the polaron structure. Those peaks can confirm the existence of PANI inside the composite. Each sample was analyzed three times (RDS: ± 001289–000112). The averaged values from three parallel measurements of the individual samples are already shown in the table. Values of elements are given in percent by weight (% m/m).

### XPS study

X-ray photoelectron spectroscopy (XPS) was applied to characterize further the chemical composition, the oxidation state of elements, and functional groups. According to results obtained, rGO/Cu MOF sample exhibited typical peaks for the organic frameworks (C1s, O1s, and N1s signals) and Cu2p_3/2_ signal centered at ca 934.4 eV corresponding to Cu^2+^ species (Fig. 3b). The oxidation state of copper also confirms the satellite feature at ca 937–946 eV typical for Cu^2+^ state (Fig. S2a). Similarly, manganese depicted a satellite feature at ca 646 eV confirming the Mn^2+^ state (Fig. S2c). The presence of C1s signal confirms the presence of rGO at ca 284.1 eV corresponding to sp^2^ carbon and satellite feature (π–π*) and ca 290.6 eV attributed to delocalized conductive π electrons. The other C1s signals, namely sp^3^ (284.6 eV), C–O (285.9 eV), C=O (287.40 eV), and OC=O (288.60 eV), could be attributed to the organic frameworks and some oxidation on the surface. After coating by PANI, the signal of C1s changed only slightly, more likely due to the low PANI concentration in the composite, which is related to the coating thickness (Fig. S3). The thin PANI coating indicates the minimal increase of nitrogen content, which could serve as a marker for PANI coating. In the case of complete coverage with the thick PANI layer, the ratio N/C should be close to 1/6^35^. This is not the case indicating a thin layer coating by PANI. Typical signals of N1s were detected at ca 399.3 eV that corresponds to –NH– and N1s. The signal at ca 401.2 eV corresponds to –N^+^ species indicating that PANI is in the form of emeraldine salt (Fig. S4). The presence of the ClO4^−^ (Cl2p) anion was detected in the spectrum at 207.2 eV, which can be explained by using an HClO_4_ solution to polymerize aniline^36–38^. Those results confirmed the successful synthesis of composites made of Mn/Cu-MOFs, rGO, and PANI.

### Elemental analysis

After the in-situ polymerization of aniline on rGO/Mn-MOF (**M1**) and rGO/Cu-MOF (**M2**) to make rGO/Mn-MOF@PANI (**M1P**) and rGO/Cu-MOF@PANI (**M2P**), the elemental analysis was carried out to find the different elements and their distribution in the composites before and after the reaction. The results obtained are shown in Table 1. As can be seen from the table, C, H, and N are the main components of samples. During the oxidization via modified Hummer’s method, KNO_3_ was reacted with graphite to transfer it to GO, which led to the N atom's existence in M1 and M2. After being supported with PANI, the percentage of N in the composites was increased. Among other things, M2P contains a higher concentration of N than M1P. Those increases of N% showed that PANI was supported on the structure of M1 and M2. Moreover, the difference in N percentage change of M2P was higher than M1P. It can be explained due to the catalytical properties of copper during the reaction. Copper compounds were reported to be an efficient catalyst for PANI, and it may lead to a higher amount of PANI being formed inside the M2P^39,40^.

**Table 1:** The elemental composition of prepared hybrids as determined by EDX-XRF method.

Samples	C (%)	H (%)	N (%)
M1	72.94 ± 0.15	0.95 ± 0.001	0.36 ± 0.01
M2	59.64 ± 1.31	0.74 ± 0.02	0.15 ± 0.01
M1P	67.99 ± 1.00	1.23 ± 0.02	0.80 ± 0.02
M2P	72.25 ± 0.43	1.33 ± 0.05	1.62 ± 0.25

The chemical compositions of the sample metal components were further determined using EDX-XRF (Detailed information is shown in Table 2). The materials will be calculated to determine the metal component percentages in the composites. It can prove that after the polymerization process, the Mn/Cu-MOFs remained stable in the composite. Furthermore, the existence of Cl can also verify the existence of PANI. During the polymerization process in HClO_4_, Cl was captured on the polymer chains, leading to its percentage appearance.

**Table 2:** Element components in the partial composites.

Samples/elements	Basic matrix (%)	Mn (%)	Cu (%)	Cl (%)
M1	95.9	0.3750	–	~ 0
M2	93.7	–	0.9655	~ 0
M1P	96.5	0.4212	–	0.1690
M2P	91.1	–	0.5942	0.1640

% m/m—percentage of weight.

*PANI* basic matrix: CHNO (polymer), *MOF* basic matrix: C (carbon).

The pristine MOFs powders have low conductivity (10^–12^–10^–14^ S cm^−1^)^41,42^. Hence, the samples’ conductivities were tested to decipher the improvement of PANI on the composites. The resulting composite of **M2** contained Cu-MOF showed higher electrical conductivities (4 × 10^–2^ S cm^−1^) than the composite of **M1** (3.3 × 10^–2^ S cm^−1^). Hence, after being supported with PANI, the composite's conductivities were increased to 2.8 × 10^–1^ S cm^−1^ (**M1P**) and 3.7 × 10^–1^ S cm^−1^ (**M2P**). The graphite sheets of the composite after the polymerization processes contained PANI on their surface tightly. Additionally, the conductivity of the composite could be increased due to the intact contact of PANI on the rGO/MOF conducting surface.

### Morphology characterizations

Figure S1 shows the SEM images of the synthesized Mn/Cu-MOFs. The prepared MOFs particles appeared to have regular and ordered structures, confirming the synthesis process's success. The SEM image of Mn-MOF particles received (Fig. S1) showed the uniform with microcrystals structures.

**Figure 4:**
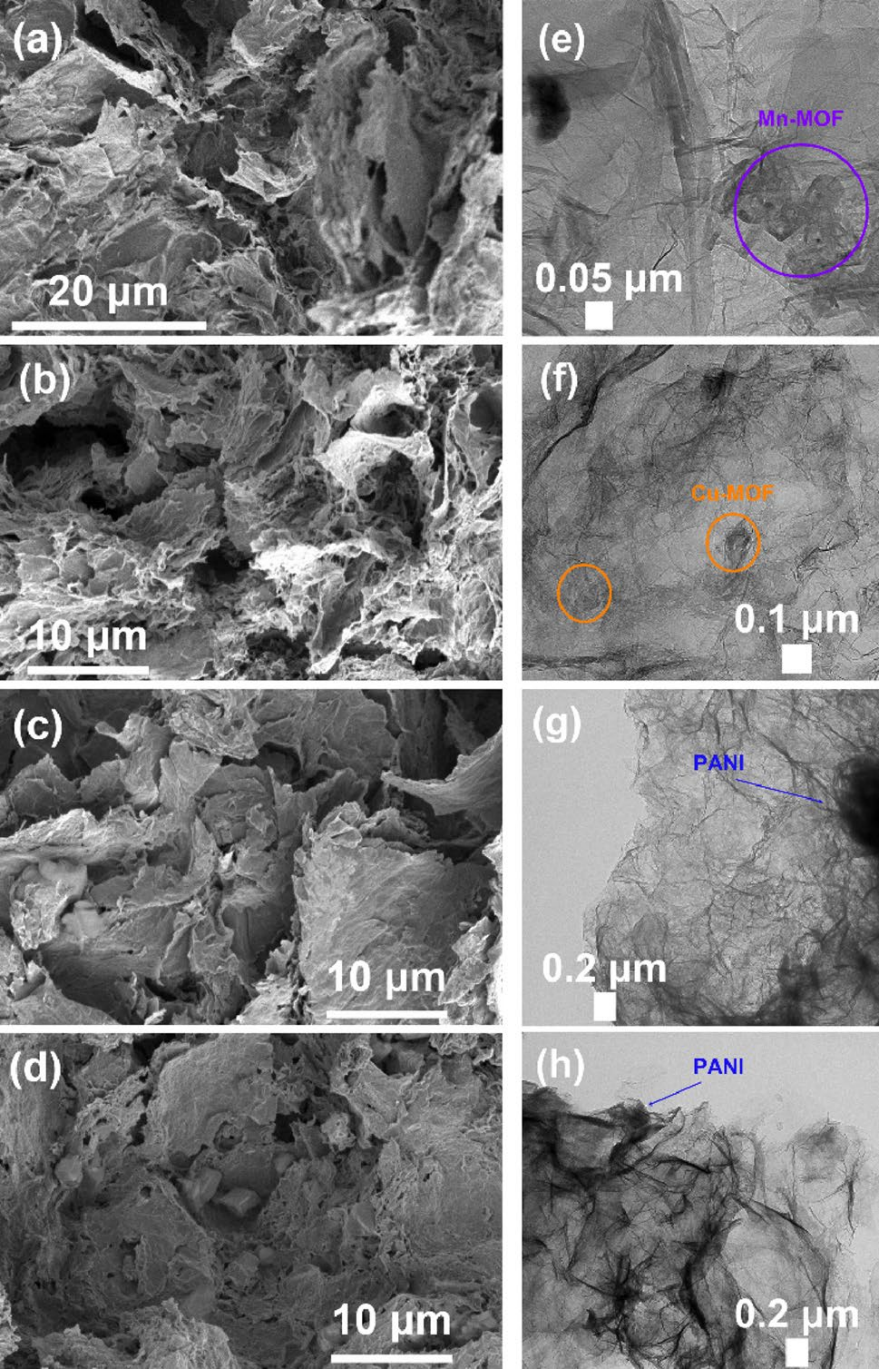
SEM images of (**a**) M1; (**b**) M2; (**c**) M1P and (**d**) M2P; TEM images of (**e**) M1; (**f**) M2; (**g**) M1P and (**h**) M2P.

Figure 4 shows the morphology of composites M1, M2, M1P, and M2P obtained via hydrothermal reaction in the autoclave. After being synthesized via the hydrothermal reaction, GO was reduced to rGO, forming the carbon graphene sheet. Those sheets contain the hydrophobic basal plane and the hydrophilic edge, which can be seen in Fig. 4. During the sheet forming reaction, Mn/Cu-MOF was captured inside the structures of those composites. According to the TEM images, MOFs particles are located inside the structures of M1 and M2 (Fig. 4e,f). Figure 4 also shows the composites' highly porous structure, facilitating access to electrolyte ions, enhancing interaction with the material surface, and activating electrochemical reactions^22^. The morphology of M1P and M2P changes markedly after their modification with PANI: PANI is distributed on or intercalates between the surfaces of rGO sheets and the MOF particles (Fig. 4g,h). Thus, PANI's addition into the composites may enhance the pseudo-capacitance of M1P and M2P, hence leading to their higher electrochemical performance.

### Electrochemical characterizations

**Figure 5:**
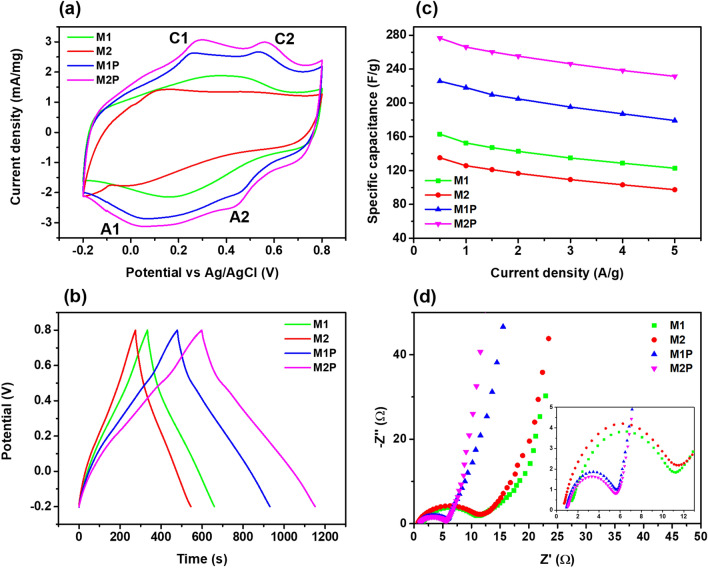
Three-electrode system performance of (**a**) cyclic voltammetry at 10 mV/s, (**b**) charge–discharge plots at 0.5 A/g, (**c**) specific capacitance calculated from GCD curves, and (**d**) electrical impedance.

To understand the operating performance of electrode materials, we compressed the composites' slurry on the titanium mess and analyzed their electrochemical properties. (details are in supplementary information). The SC electrode characteristics are generally measured and deciphered using the cyclic voltammetry (CV) analysis. Figure 5 compared the electrochemical properties of the electrodes made of those composites. We used the typical tree-electrode system to CV test in a fixed potential window of − 0.2 to 0.8 V using 1 M H_2_SO_4_ as the electrolyte^43^. The samples showed that the response currents of M1 and M2 exhibited redox peaks caused by the electrode materials and carbon’s faradic reactions. They can be expressed as follows:

1 $${\text{M}}\left( {{\text{C}}_{{8}} {\text{H}}_{{4}} {\text{O}}_{{4}} } \right)\left( {{\text{H}}_{{2}} {\text{O}}} \right)_{{2}} + {\text{C}}^{ + } + {\text{e}}^{ - } \leftrightarrow {\text{MC}}\left( {{\text{C}}_{{8}} {\text{H}}_{{4}} {\text{O}}_{{4}} } \right)\left( {{\text{H}}_{{2}} {\text{O}}} \right)_{{2}}$$

where M denotes the metal ion of Mn and Cu from the MOFs compound^44^. The CV spectra of samples at different scan rates from 10 to 100 mV s^−1^ can be found in Fig. S5. Notably, the electrodes made of M1P and M2P showed higher current density than M1 and M2, indicating that their capacitances were enhanced. We can see two pairs of the redox peaks showed in the CV curves of M1P and M2P, which are related to the surface reactions of PANI among the reduced state, the partially oxidized state, and the complete oxidation states, respectively^45^. The appearance of those redox peaks could be explained through the insertion and desertion of SO_4_^2−^ ions (doping) in PANI according to Eq. (2):

2 $$\left( {{\text{PANI}}} \right)_{n} + ny \; {\text{SO}}_{{4}}^{{{2} - }} \to \left[ {{\text{PANI}}^{{{\text{y}} + }} {\text{SO}}_{{4}}^{{{2} - }} } \right] + ny\;{\text{e}}^{ - }$$

where *y* is the doping degree, defined as the ratio between the number of charges in the polymer and the number of monomer units^46^. Additionally, PANI has a good conductivity property. During the working process, under the current flow, PANI can be easily converted between various oxidation states such as emeraldine, leucoemeraldine, and pernigraniline. The spectra of M1P and M2P showed two pairs of redox peaks related to the activity in acidic aqueous electrolytes. The first anodic peaks can be associated with doping of SO_4_^2−^ anions related to the conversion of leucoemeraldine form of PANI to emeraldine salt (C1/A1). The next peak signifies the transition of emeraldine to a fully oxidized pernigraniline state (C2/A2)^47^. The reaction mechanism of PANI salts was displayed in Fig. 1.

The explanation of specific capacitance improvements when supported with PANI into the composite structures was also displayed by the galvanostatic charge–discharge (GCD) curves. Figure 5b showed that all composites exhibited the triangle shapes when they were tested at the current densities of 0.5 A/g, which implies good electrochemical reversibility^48^. The specific capacitance value was calculated as follows:

3 $${C}_{s}= \frac{I.\Delta t}{m. \Delta V}$$

where *I* denotes the applied current (A), *Δt* is the time taken for the discharge, *m* is the mass of active electrode material, and *ΔV* is the discharge potential^22^.

The GCD curves of M1 and M2 showed that the Mn-MOF composite has a longer discharging time than the composite of Cu-MOF, which can cause a higher specific capacitance of M1 than M2. This phenomenon can be explained due to the higher numbers of different oxidation states of Mn ions. During the charge/discharge process, Mn^4+^ can be formed. They can facilitate the bulk redox reactions, enhancing the operating performance when used as a supercapacitor^48^. To understand the composites' electrochemical properties, we conducted the GCD tests of those electrodes in different scan rates from 0.5 to 5 A/g (Fig. S6). The capacitances of electrodes made of those composites decrease when the current densities increased (Fig. 5c).

It can be explained by ions' faradic reaction between the electrolyte and the electrode materials during the scanning process. At the high scanning rates, the electrolyte ions’ accessibility to the electrode materials' surface decreased. Indeed, it is proven that the capacitance of the electrode materials depends on their structure and morphologies^49^. The porous structure of the samples is beneficial for ionic conductivity, while the components of the composites can improve the ion transportability of electrodes.

Moreover, porous bridges inside the electrode allow it to overcome the limitations on both intercalation and adsorption of charged ions. The porous structure also increased the possibility of the interaction between those ions, which led to a better opportunity for reactions^49^. Considering these reasons, we can assume that an increase influenced the electrolyte ion's diffusion into the electrode matrix in the sweep rate. The electrolyte ions did not interact with the electrodes for a long time and could not reach deep pores. Consequently, this led to a decrease in the specific capacity of the composites.

However, as we described before, Cu-MOF catalyzed in-situ aniline polymerization during the polymerization process, leading to higher PANI in M2P than M1P. PANI is generally considered to enhance the electrochemical performance of carbon-rich due to its high pseudo-capacitance^45^. The M1P and M2P composites showed their specific capacitance of 225.8 and 276.6 F/g at 0.5 A/g, respectively, while the values of M1 and M2 are 163.2 and 135.2 F/g. It can be explained by a higher PANI amount inside M2P than in M1P and better distribution of PANI inside of MP2, which demonstrated pseudo-capacitance improvement of M2P. The specific capacitance enhancement of M1P and M2P at high current densities can also be attributed to the synergetic effect of pseudo-capacitance and EDLC between PANI and the Mn/Cu-MOFs particles inside the structure. The MOFs usage in the electrochemical application can provide good EDLC properties when they were combined with rGO^15^. At the same time, PANI, being a conductive polymer, can promote electrical contacts between structures within M1P and M2P. It can facilitate diffusion contact between electrolyte ions during scanning, causing high pseudo-capacitance results of those materials^31^. Consequently, the combination of PANI and MOFs particles consequently helps to increase the specific capacitance results at high scan rates.

Next, we conducted electrochemical impedance studies (EIS) to decipher the ion transport mechanism. The positive of the improvement of the materials can be identified via the EIS results. Figure 5d shows that the composites' Nyquist plots have the semicircles in the high and mid-frequency regions from the real axis, most likely due to the charge transfer resistance (R_ct_) between the electrode and electrolyte interfaces^50^. The values of R_ct_ can be determined from the radius of the semicircles. Comparing with the R_ct_ values for M1 (9.86 Ω) and M2 (10.83 Ω), the lower R_ct_ values of M1P (4.74 Ω) and M2P (4.53 Ω) suggest the ideal capacitive behavior due to the better ion diffusion path which can facilitate the diffusion rate when PANI was supported to the composites^51^. The oblique line’s slope in low-frequency regions relates to the ions diffusion rate in the electrolyte. Figure 5d shows that the composites of M1P and M2P exhibited a more straight-line long imaginary axis compared to M1 and M2, which can indicate that lower diffusion resistance of the electrolyte ions in their working process^52^.

Although MOFs are novel developed porous materials that can be easily adjusted by altering their bridging organic ligands, the poor conductivity has always been the notable hindrance to promoting the electrochemical performance of most MOFs applied on SCs. However, due to their excellent specific surface areas and easily adjustable pore environments, MOFs still have many potential opportunities to improve their working performance. Furthermore, according to their metal and carbon sources, MOFs can have various morphologies, which can facilitate their cooperation with conducting polymer to fabricate the high working performance composite materials^17^. The specific capacitance of this study and other composite materials for supercapacitor electrodes are compared and shown in Table S1. Compared to the published results, rGO, Mn/Cu-MOF, and PANI composites showed excellent specific capacitances at 0.5 A/g. The high working performance of those composite materials is mainly attributed to PANI's pseudocapacitance and the combination of rGO and Mn/Cu-MOF. In our previous study, the specific capacitance of rGO/Zn-MOF@PANI at 0.5 A/g was 253.35 F/g. The electrode materials derived from rGO, MOFs, and PANI composite may present ideal porous structures for ease-diffusion of electrolyte ions. The results shown in Table S1 also exhibit the change of the specific capacitances at the same testing conditions related to the structure of metal ions inside MOFs’ structures.

### Cyclic stabilities

**Figure 6:**
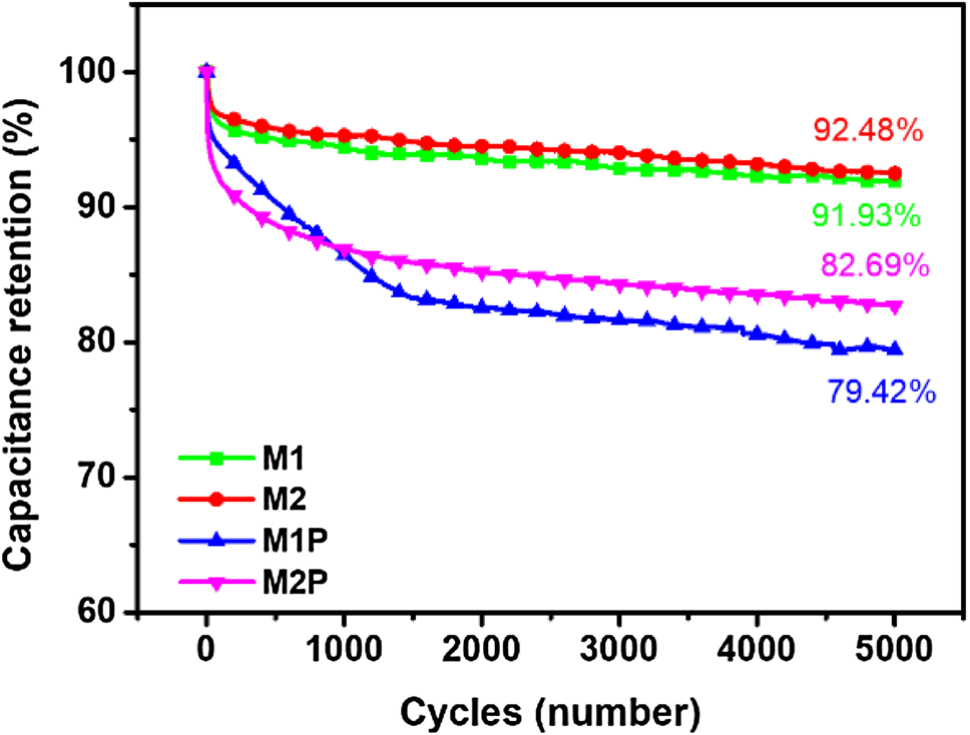
The cyclic capacity retention during 5000 cycles at 1 A/g.

To study the retentions of composites, we tested the two-electrode symmetrical SC. Figure 6 shows the samples' cycling stability performance at the applied current density of 1 A/g for 5000 cycles. The composite of M1 and M2, which contain only Mn/Cu-MOF and rGO, shows good cycle stability with capacitance retention of approximately 92%. However, M1P and M2P retentions show a visible decrease for the first 1500 cycles due to PANI degradation during the charge/discharge process^53^. Then, the retention ability is stabilized at the capacitance retentions of 82.69% and 79.42% for M2P and M1P, respectively. The better retention of M2P to M1P can be attributed to the former’s higher PANI percentage contained inside.

The stabilities of the composites were further confirmed by applying the potential scan rate at 10 mV s^−1^ for M1, M2, M1P, and M2P electrodes (Fig. S7a). There are slight changes in the shapes of the cyclic voltammogram of the electrode’s materials. The significant changes of M1P may be due to the deformation of the PANI composite on the surface. Two peaks appeared on the curve of M2P show that the redox reaction of PANI converted between various oxidation states such as emeraldine, leucoemeraldine, and pernigraniline. The charge–discharge plots of the composites also show their high capacitance retentions after 5000 cycles of stability tests (Fig. S8). After stability tests, the capacitive behavior of composites was shifted and can be seen via the semicircle of R_ct_ in Fig. S7c. The reduced R_ct_ of M2P showed a better ion diffusion rate inside the composite structure^51^. Hence, the oblique line’s slope in low-frequency regions relates to the ions diffusion rate of M1P was shifted and show the diffusion of the ions inside the composite now decreased. It also explained the R_ct_ increase of M1P^52^. The excellent stability of M2P may be due to the catalytical phenomenon of Cu-MOF on the in-situ growth of PANI on rGO sheets. The amount and stable PANI synthesized in M2P can facilitate efficient electron transfer. Hence, it can help PANI prevent structural degradation and deformation.

Figures S9 and Fig. S10 displayed the surface morphologies of the electrodes materials before and after stability tests, respectively. As we can see in Fig. S9, after the electrodes fabrications process, PTFE polymers appeared and covered the surface of the rGO matrices of the composites. PTFE plays the binder's role, which can help the composites to maintain their structure on the titanium meshes. However, due to the coverage, the other components cannot be seen clearly via the SEM images of the surface. After 5000 cycles, the amounts of PTFE on the surfaces were reduced (Fig. S10). Hence, the IR spectra of the electrode’s materials shown in Fig. S11 proved the existence of the components on the structure before and after 5000 cycles tests. Due to the addition of PTFE, the C-F asymmetric stretching peaks at 1151.29 cm^−1^ and symmetric stretching peaks at 1209.15 cm^−1^ appeared on every sample^54^. The spectra of all samples did not show the change before and after the stability tests. The high-intensity peaks at 1588 cm^−1^ related to the asymmetric vibration of –COO groups that confirmed that Mn/Cu-MOFs have remained inside the composites after the stability test.

Although PANI is a promising material, it still has drawbacks that can restrain its application on SCs. As mentioned in the paper, PANI showed substantial degradation during the doping/undoing process, limiting the stability of SC for long cycles use^6^. Furthermore, during the charge/discharge cycle process, the electronic conductivity of PANI tends to decrease due to the change of their molecular structures while the faradaic reactions are conducted on the surface of the PANI chains^55^. The number of charges can be stored or released when a PANI molecule switches between the highly and poorly conducting state via the electrochemical redox reactions. After a certain number of working cycles, PANI will be degraded. Hence, it leads to decreased electrical current carrying capacity and storage capacitance of applied SCs^56^.

The combination of PANI with other materials to form the composites in this study aims to solve those problems mentioned. The choice of the constituents of composites will cause an effect on their conductivity, surface areas, chemical endurance, and mechanical durability of SCs’ electrodes. Those characteristics of electrodes will directly influence the capacitance of an SC. Both capacitance and stability are vital for the working of SC. An SC has high specific capacitance but cannot work for a long time, or a stable SC can keep their retention for an extended period, but low specific capacitance; neither of their application is limited. There is no priority in this matter. Our group is doing our best to optimize the SC by combining two vital factors.

In this study, there are only two kinds of MOF components composited with PANI. The obtained results can show the improvement in the specific capacitance of SCs after conjugating the composite with PANI. To increase the electrochemical performance and make the tuning conclusion, more samples should be prepared to show the scientific evidence. The treatment of the MOF materials or the composite after synthesizing can make the working performance change.

## Conclusion

In summary, composites made of rGO, Mn/Cu-MOF were fabricated and supported with PANI to be used as electrode materials for SCs. It was found that composite containing Cu-MOF acts as a catalyst for aniline polymerization, which leads to a higher concentration of PANI, thus improving the working performance of the electrode. This study agrees well with reported literature results that the introduction of PANI into the composites increases the pseudo-capacitance, thus enhancing the electrode materials' working performance. Besides it, composites also exhibited cycle stability. The results obtained confirm the high potential of materials based on MOFs, rGO, and conductive polymers as components of electrode materials for SCs.

## Supplementary Information

Supplementary Information.
