# Supplementary material for: Electrochemical performance of composite electrodes based on rGO, Mn/Cu metal–organic frameworks, and PANI
Source: Sci Rep. 2022 Jan 13;12:664. doi: 10.1038/s41598-021-04409-y (PMC8758744; doi:10.1038/s41598-021-04409-y)
Supplement: Supplementary file 1 — Supplementary Information. [file 41598_2021_4409_MOESM1_ESM.pdf]

Supplementary information

Electrochemical performance of composite electrodes based on rGO, Mn / Cu metal-organic frameworks, and PANI

Quoc Bao Le[a,b,*], Thanh-Huong Nguyen[c], Haojie Fei[a], Constantin Bubulinca[a], Lukas Munster[a], Nikola Bugarova[d], Matej Micusik[d], Rudolf Kiefer[b], Tran Trong Dao[e], Maria Omastova[d], Natalia E. Kazantseva[a], Petr Saha[a]

[a] Centre of Polymer Systems, Tomas Bata University in Zlín, Tř. T. Bati 5678, 760 01, Zlín, Czech Republic.

[b] Conducting Polymers in Composites and Applications Research Group, Faculty of Applied Sciences, Ton Duc Thang University, Ho Chi Minh City, Vietnam

[c] NTT Hi-Tech Institute, Nguyen Tat Thanh University, Ho Chi Minh City 72820, Vietnam.

[d] Polymer Institute, Slovak Academy of Science, Dubravska cesta, 9, 845 41 Bratislava, Slovakia.

[e] Division of Modeling Evolutionary Algorithms Simulation and Artificial Intelligence, Faculty of Electrical & Electronics Engineering, Ton Duc Thang University, Ho Chi Minh City, Vietnam.

KEYWORDS supercapacitor, metal-organic framework, reduced graphene oxide, polyaniline.

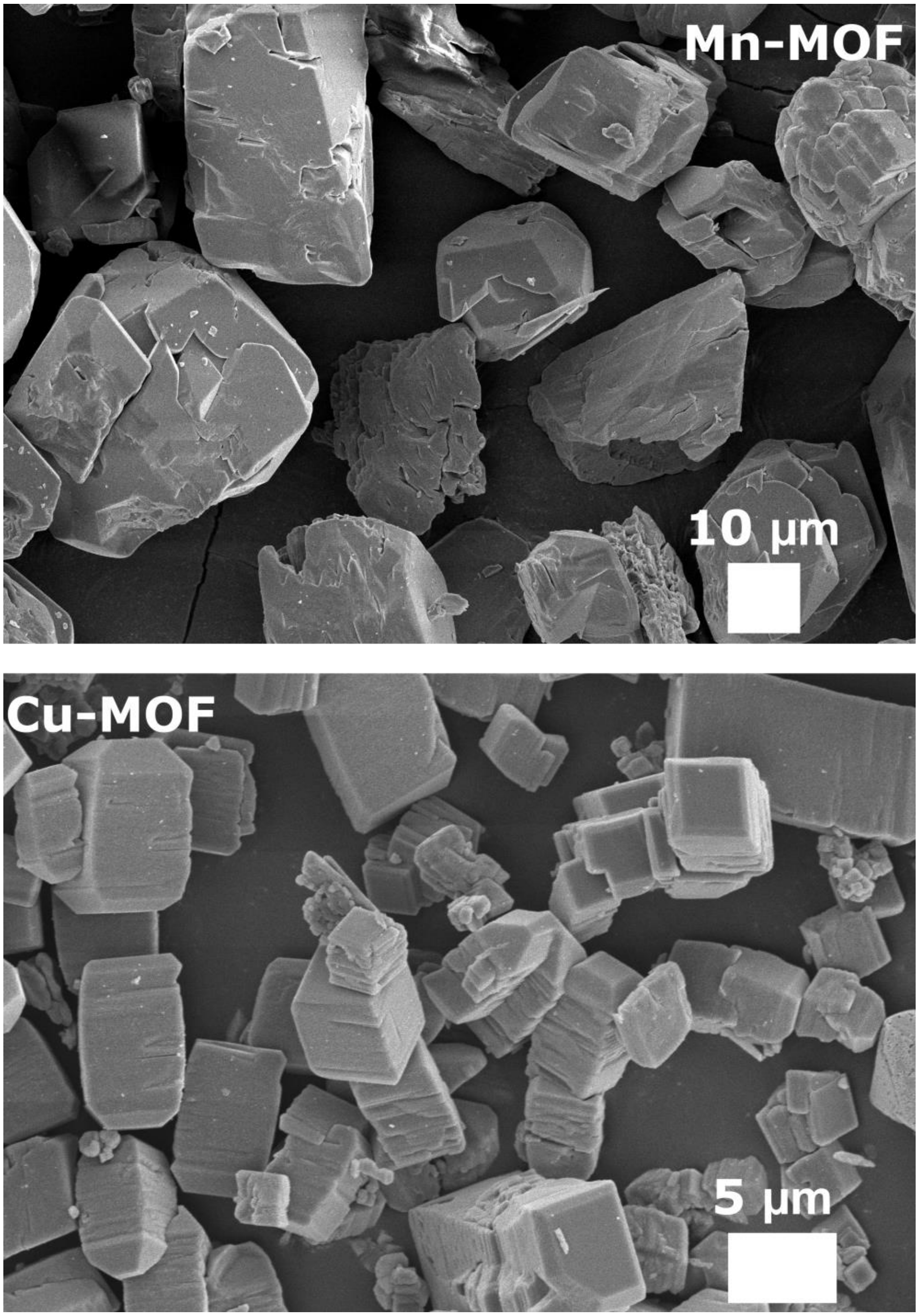

Figure S1. SEM images of Mn-MOF, and Cu-MOF.

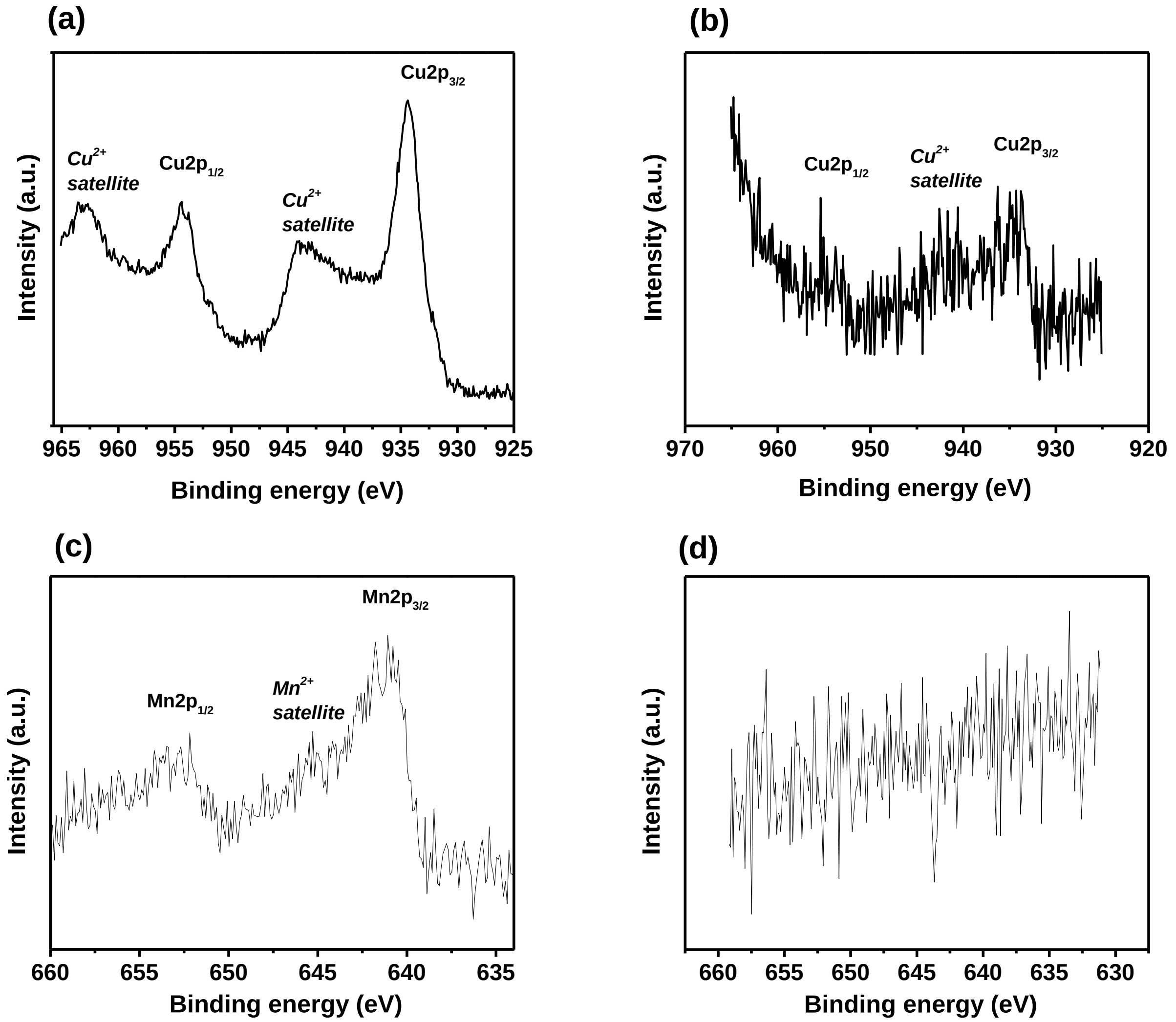

Figure S2. XPS of Cu2p region of a) rGO-Cu-MOF, b) rGO-Cu-MOF@PANI and of Mn2p region of c) rGO-Mn-MOF, d) rGO-Mn-MOF@PANI. [Thermo Fisher Scientific, Thermo Avantage software 5.9922, XPS knowledge database].

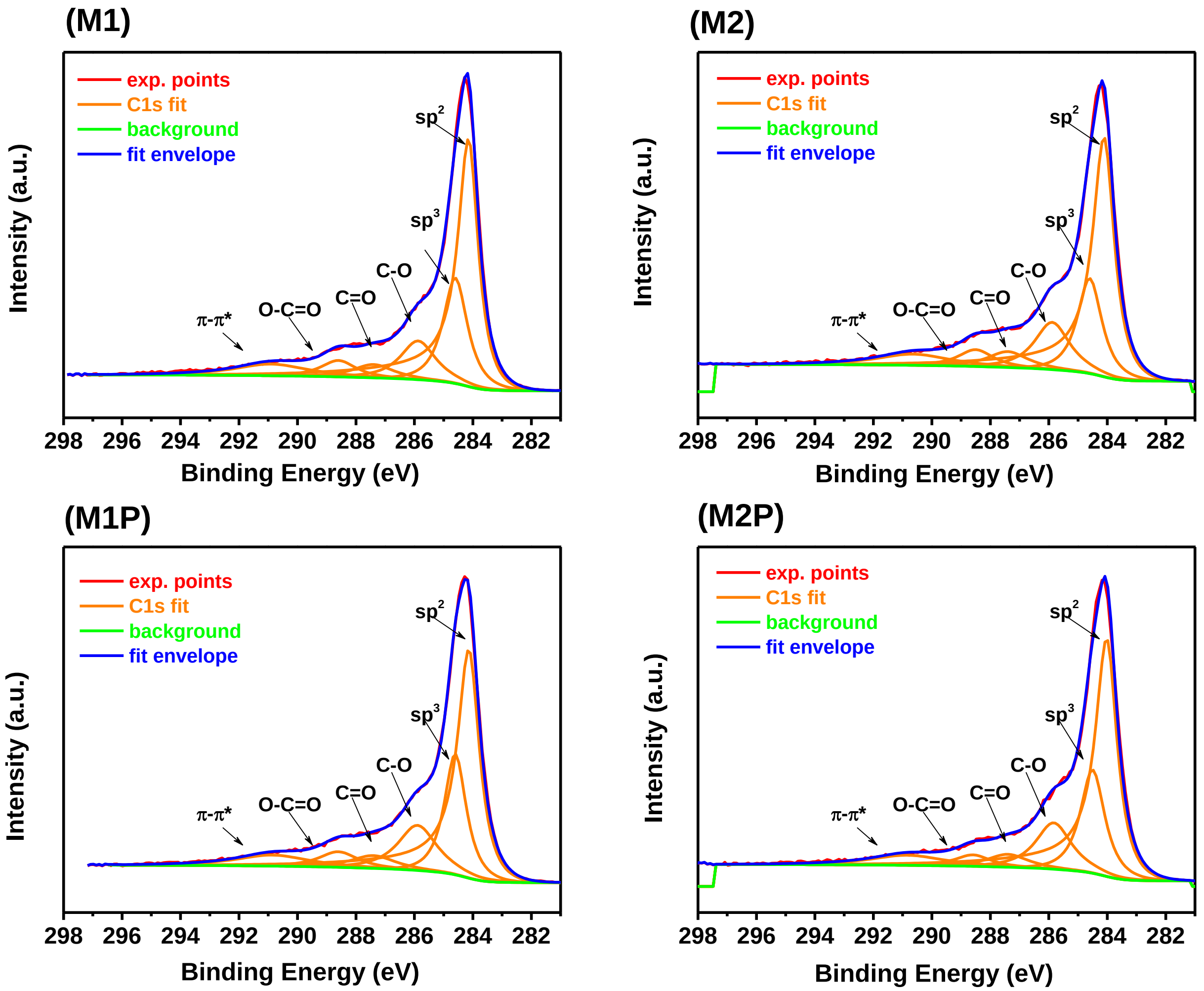

Figure S3. XPS of C1s region of M1, M1P, M2 and M2P.

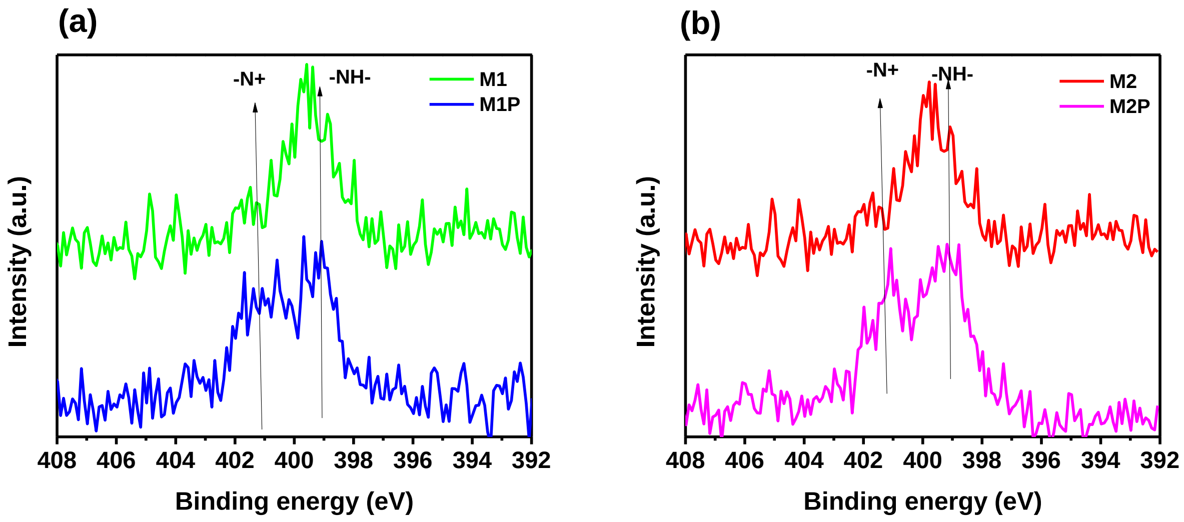

Figure S4. XPS of N1s region of a) M1 and M1P, b) M2 and M2P.

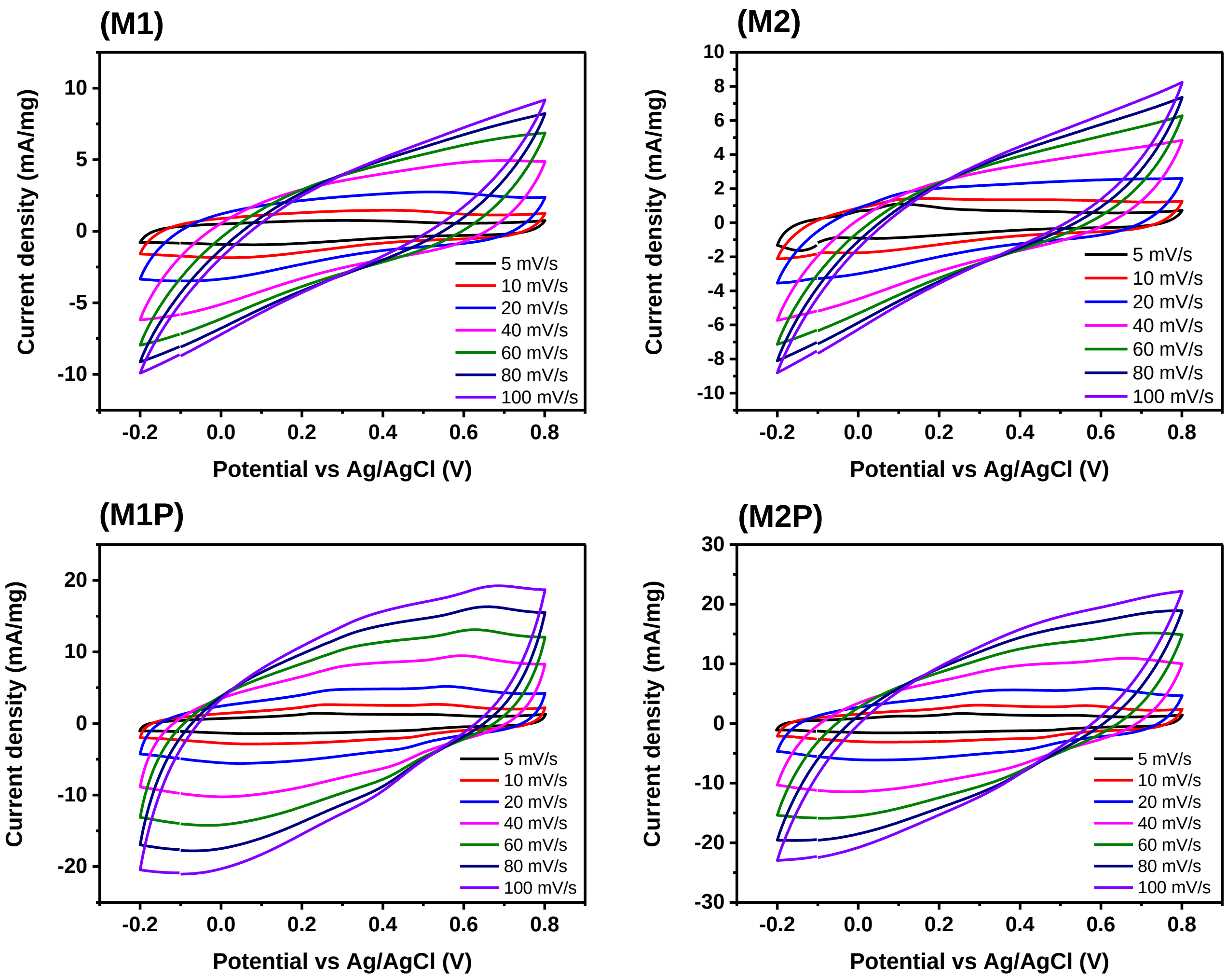

Firgure S5. Cyclic voltammetry of M1, M2, M1P and M2P.

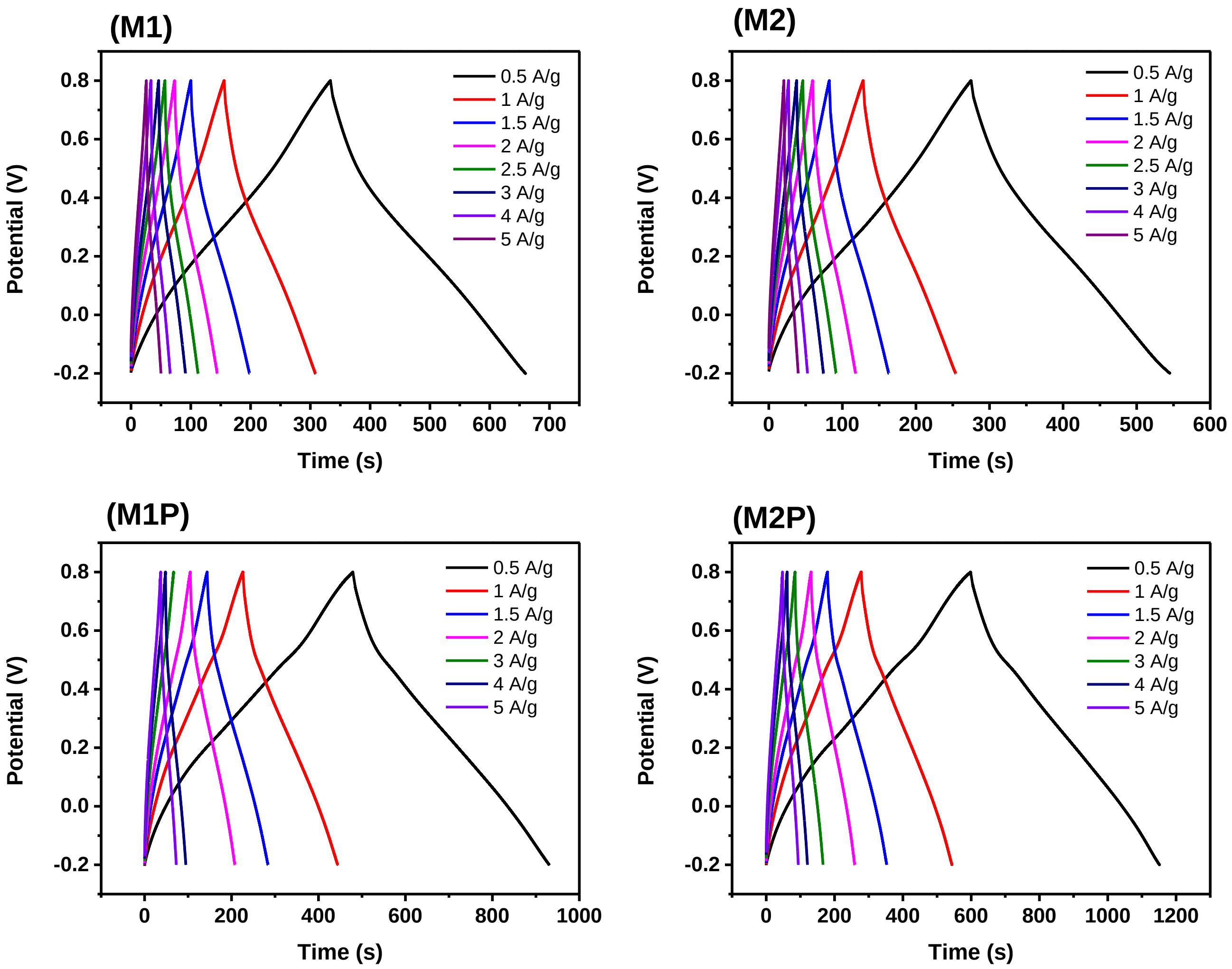

Figure S6. Charge-discharge plots of M1, M2, M1P and M2P.

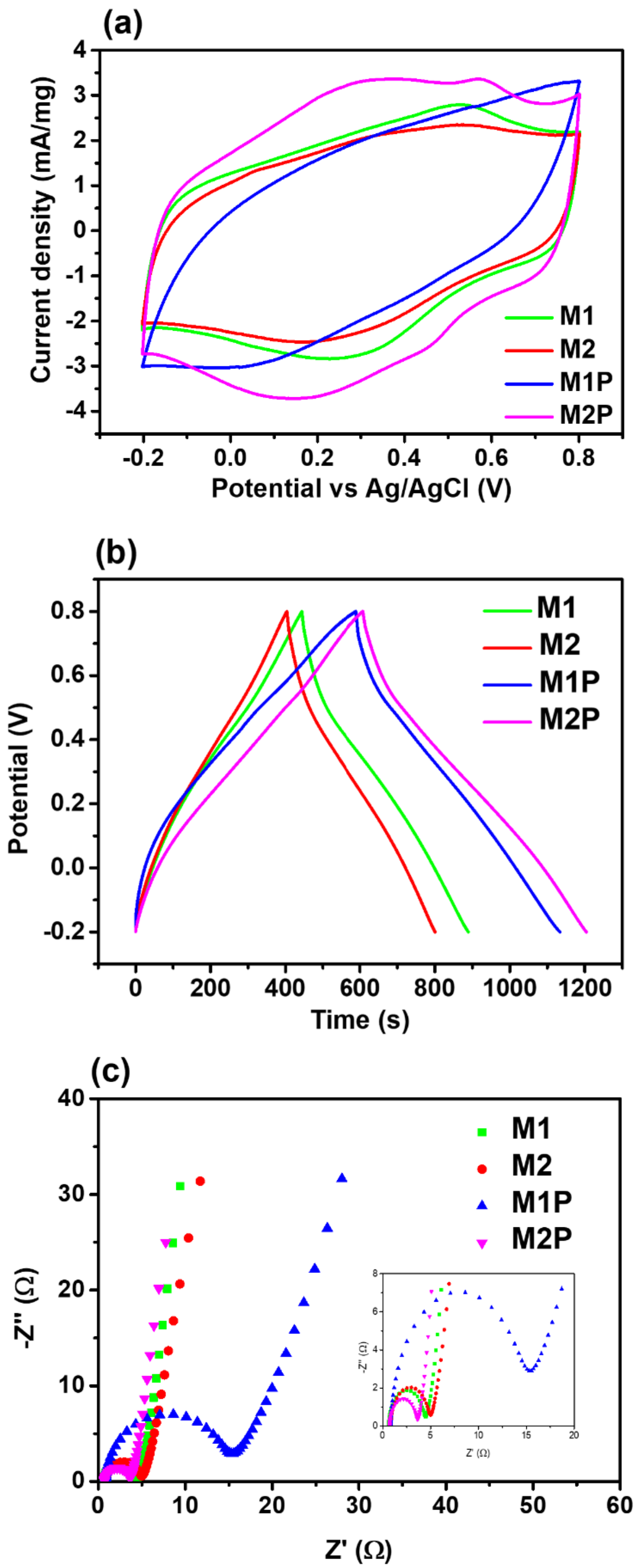

Figure S7. Three-electrode system performance of (a) cyclic voltammetry at 10 mV/s, (b) charge-discharge plots at 0.5 A/g of all composites, and (c) their EIS plot after 5000 cycles stabilities test.

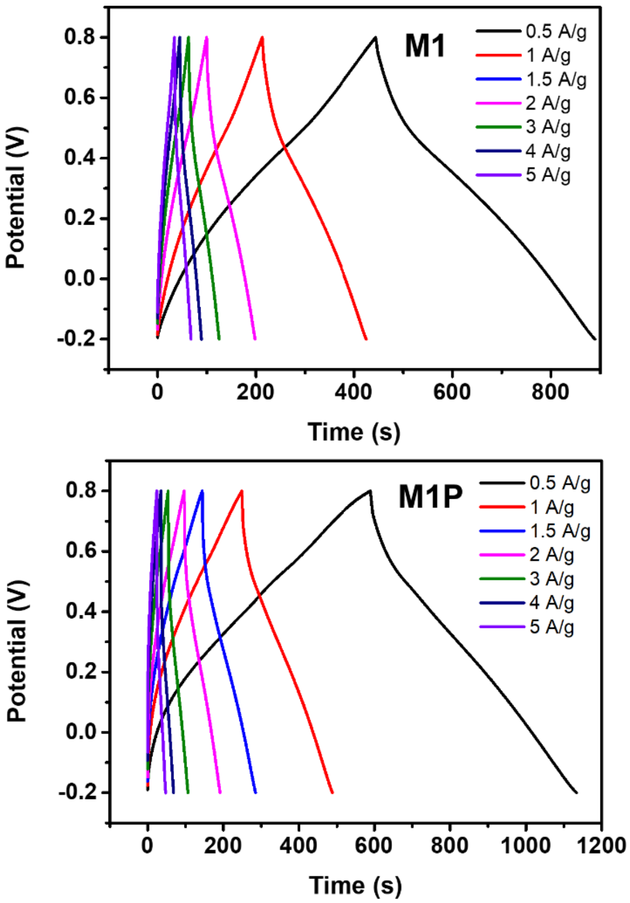

Figure S8. Discrete plots at different current densities using three-electrode system of the composites supercapacitor electrode materials.

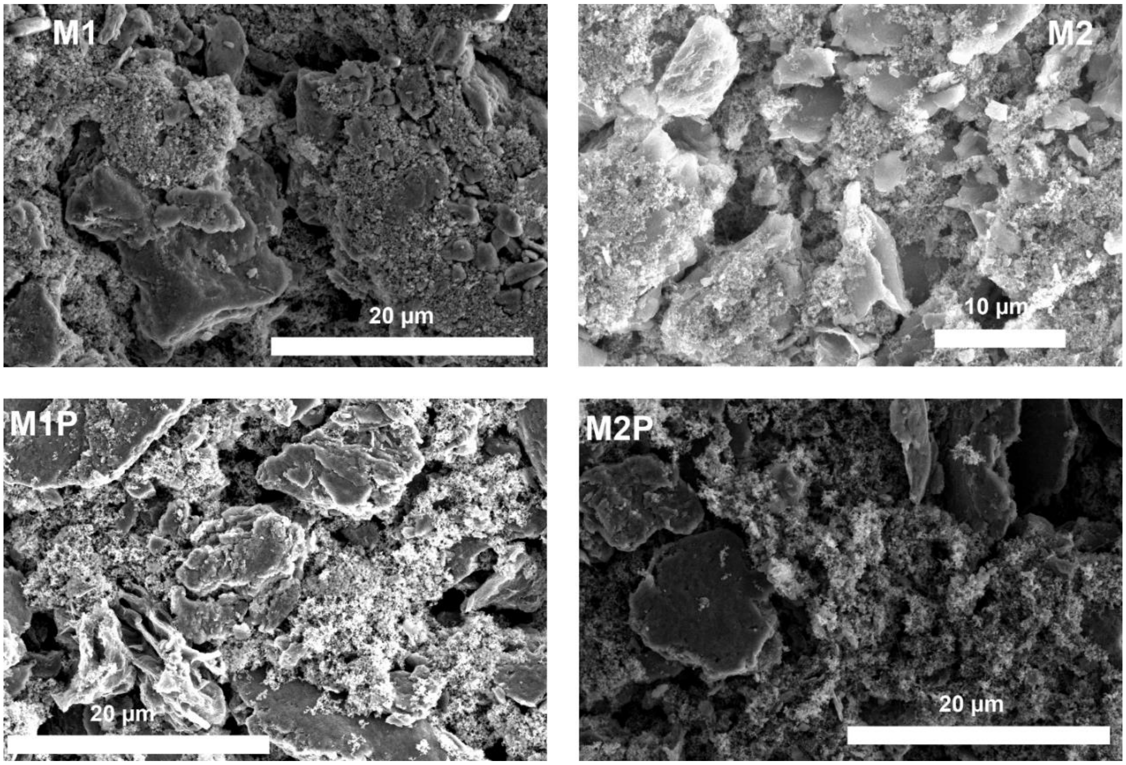

Figure S9. SEM images of the surfaces of electrodes made of M1, M2, M1P, and M2P before stability tests.

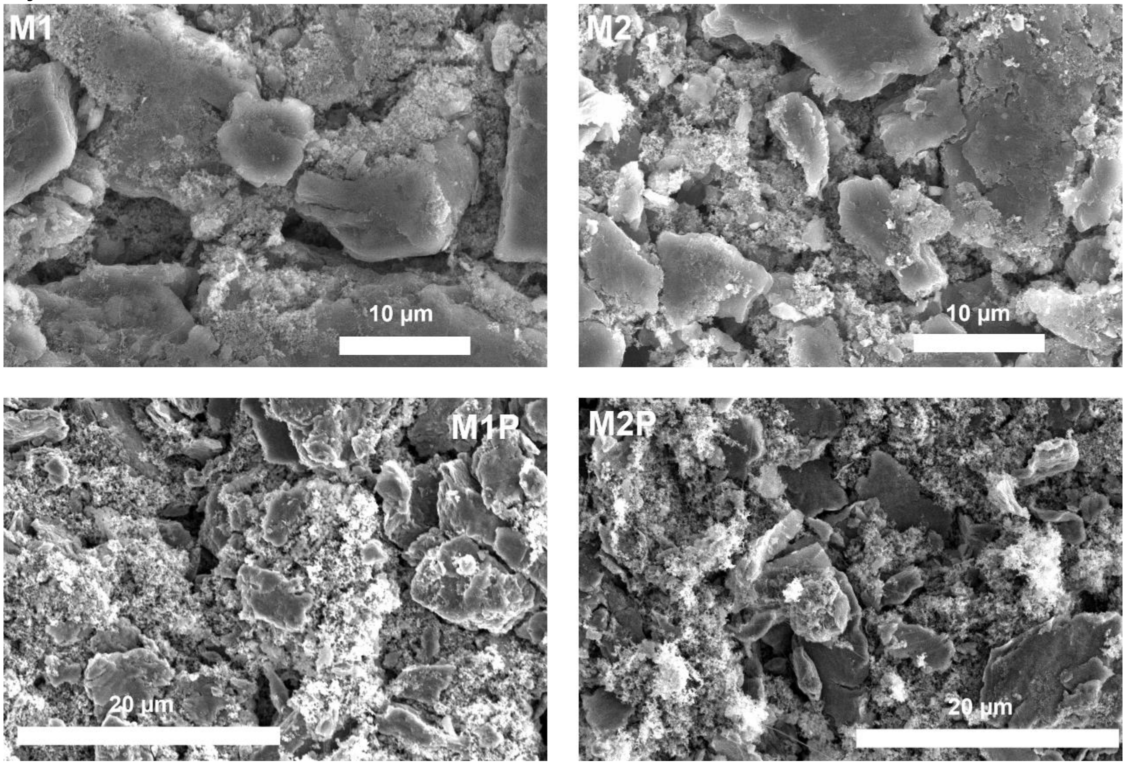

Figure S10. SEM images of the surfaces of electrodes made of M1, M2, M1P, and M2P after stability tests.

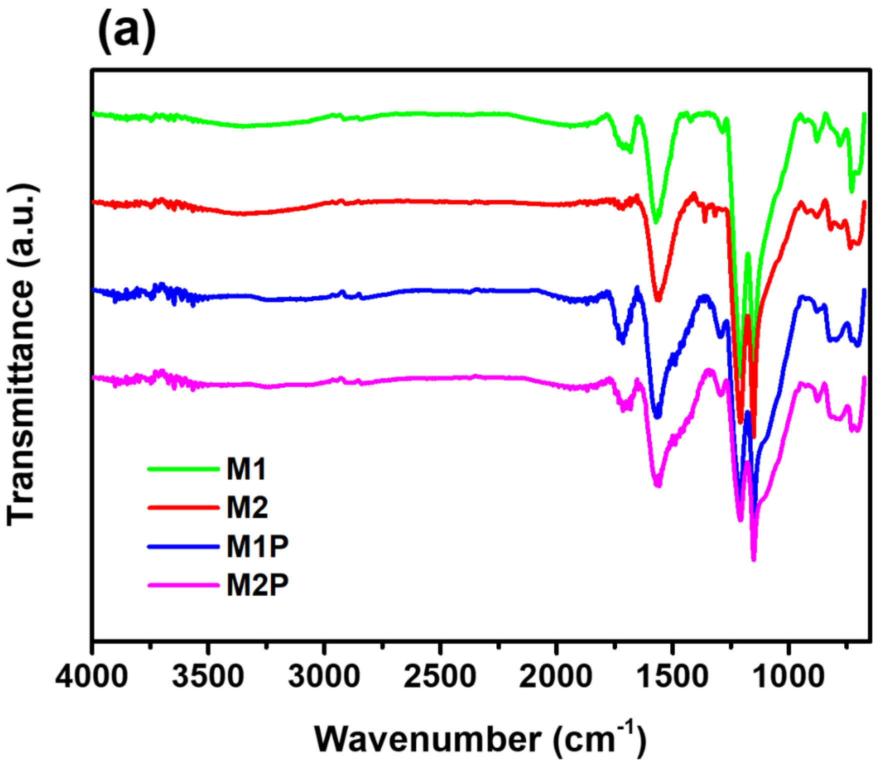

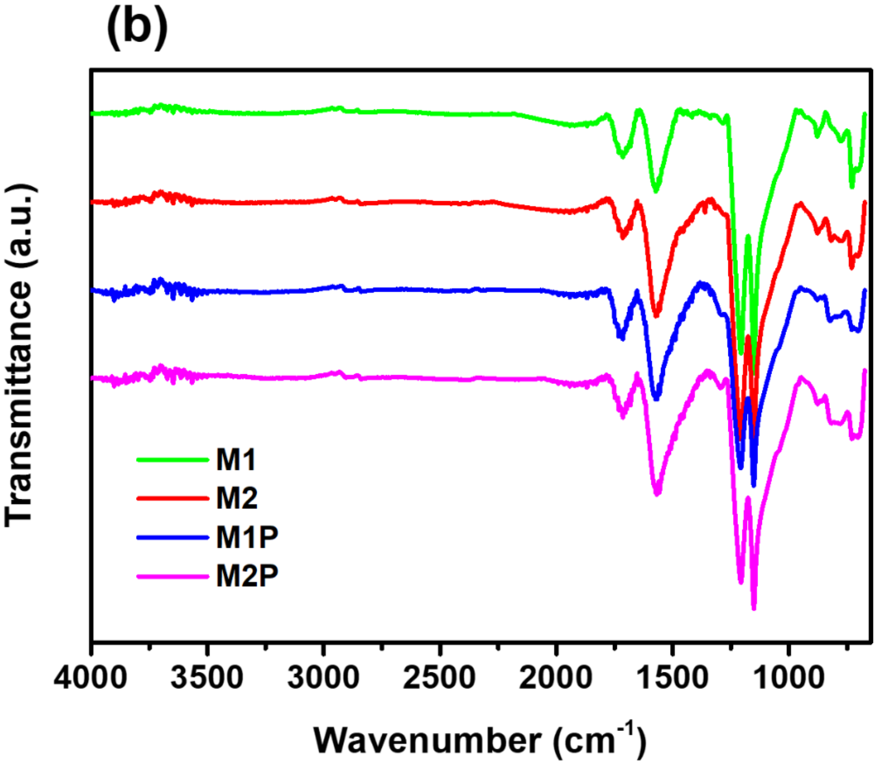

Figure S11. FTIR spectra of electrodes made of the composites **(a)** before and **(b)** after stability tests.

Table S1: Comparison of the specific capacitance of composites obtained in this study with reported literature.

No.	Compound	Electrolyte	Specific capacitance	Scan rate	Reference No.
1	rGO/Cu-MOF	1 M KCl	44.6 mF/cm^2	5 mV/s	[1]
2	Cu-MOF@CNT	1 M Na_2SO_4	380 F/g	1.6 A/g	[2]
3	CNTs@Mn-MOF	1 M Na_2SO_4	202.8 F/g	1 A/g	[3]
4	rGO/$ZnMn_2O_4$@PANI	1 M H_2SO_4	297.80 F/g	0.2 A/g	[4]
5	Co/Mn-MOFs@Rice Husks	2M KCl	30.3 F/g	10 mV/s	[5]
6	rGO/Zn-MOF@PANI	1 M H_2SO_4	253.35 F/g	0.5 A/g	[6]
7	rGO/Mn-MOF@PANI	1 M H_2SO_4	225.79 F/g	0.5 A/g	This study
8	rGO/Cu-MOF@PANI	1 M H_2SO_4	276.58 F/g	0.5 A/g	This study

References

(1) Wang, Y. F.; Yang, S. Y.; Yue, Y.; Bian, S. W. Conductive Copper-Based Metal-Organic Framework Nanowire Arrays Grown on Graphene Fibers for Flexible All-Solid-State Supercapacitors. *J. Alloys Compd.* **2020**, *835*, 155238. https://doi.org/10.1016/j.jallcom.2020.155238.

(2) Ansari, S. N.; Saraf, M.; Gupta, A. K.; Mobin, S. M. Functionalized Cu-MOF@CNT Hybrid: Synthesis, Crystal Structure and Applicability in Supercapacitors. *Chem. - An Asian J.* **2019**, *14* (20), 3566–3571. https://doi.org/10.1002/asia.201900629.

(3) Zhang, Y.; Lin, B.; Sun, Y.; Zhang, X.; Yang, H.; Wang, J. Carbon Nanotubes@metal–Organic Frameworks as Mn-Based Symmetrical Supercapacitor Electrodes for Enhanced Charge Storage. *RSC Adv.* **2015**, *5* (72), 58100–58106. https://doi.org/10.1039/c5ra11597c.

(4) Le, Q. B.; Vargun, E.; Fei, H.; Cheng, Q.; Bubulinca, C.; Moučka, R.; Sapurina, I.; Tran, T. D.;

Kazantseva, N. E.; Saha, P. Effect of PANI and PPy on Electrochemical Performance of RGO/ZnMn2O4 Aerogels as Electrodes for Supercapacitors. *J. Electron. Mater.* **2020**, *49* (8), 4697–4706. https://doi.org/10.1007/s11664-020-08198-4.

(5) Kim, H.; Sohail, M.; Wang, C.; Rosillo-Lopez, M.; Baek, K.; Koo, J.; Seo, M. W.; Kim, S.; Foord, J. S.; Han, S. O. Facile One-Pot Synthesis of Bimetallic Co/Mn-MOFs@Rice Husks, and Its Carbonization for Supercapacitor Electrodes. *Sci. Rep.* **2019**, *9* (1), 1–10. https://doi.org/10.1038/s41598-019-45169-0.

(6) Quoc Bao, L.; Nguyen, T.-H.; Fei, H.; Sapurina, I.; Ngwabebhoh, F. A.; Bubulinca, C.; Munster, L.; Bergerová, E. D.; Lengalova, A.; Jiang, H.; Trong Dao, T.; Bugarova, N.; Omastova, M.; Kazantseva, N. E.; Saha, P. Electrochemical Performance of Composites Made of RGO with Zn-MOF and PANI as Electrodes for Supercapacitors. *Electrochim. Acta* **2021**, *367*, 137563. https://doi.org/10.1016/j.electacta.2020.137563.
